# Efficacy of Broadline® in cats against induced infections with developing fourth-stage larval and adult *Ancylostoma ceylanicum* hookworms^☆^

**DOI:** 10.1016/j.vpoa.2020.100025

**Published:** 2020-05-11

**Authors:** Martin Knaus, Piyanan Taweethavonsawat, Tara Cheesman, Martin Visser, Steffen Rehbein

**Affiliations:** a Boehringer Ingelheim Vetmedica GmbH, Kathrinenhof Research Center, 83101 Rohrdorf, Germany; b Parasitology Unit, Faculty of Veterinary Science, Chulalongkorn University, Bangkok, 10330, Thailand; c Boehringer Ingelheim Animal Health Australia Pty, Macquarie Park, NSW 1680, Australia

**Keywords:** *Ancylostoma ceylanicum*, Fipronil, (S)-methoprene, Eprinomectin, Praziquantel, Efficacy, Nematodes, Cat

## Abstract

*Ancylostomaceylanicum* hookworms parasitize dogs and cats in subtropical and tropical South East Asia and Pacific region, the Arab Peninsula, southern Africa and South America but cause patent infections in humans too.

To evaluate the efficacy of Broadline®, a combination product comprising eprinomectin, fipronil, (S)-methoprene and praziquantel, against developing fourth-larval stage (L4) and adult *A. ceylanicum* in cats, a blinded, induced infection study was conducted. Following oral inoculation of 24 purpose bred cats with ˜300 infectious *A. ceylanicum* larvae each, cats were allocated to one of three groups of eight animals, each which served either as untreated controls or received Broadline® (once topically at the minimum label dose of 0.12 mL per kg bodyweight) when the hookworms were L4 (five days post inoculation, dpi) or adults (25 dpi). Efficacy was determined on nematode counts following necropsy of the cats.

Fecal examination at 22 dpi confirmed that all cats of the control group and of the group to be treated when the hookworms were adult were excreting hookworm eggs (range, 100 to 1000 eggs per gram) while no eggs were recovered from the feces of the cats treated with Broadline® 5 dpi. Nematode counts established 35 dpi demonstrated that Broadline® treatment was 100% efficacious against both, developing L4 and adult *A. ceylanicum*: no hookworms were recovered from any Broadline®-treated cat while all control animals harbored *A. ceylanicum* (range, 8–35) (*p* < 0.001).

No health problems were observed throughout the study.

## Highlights

• Broadline® is highly effective against developing and adult *A. ceylanicum* hookworms in cats.
• Treatment reduces the risk of transmission to other animals and zoonotic risk to humans.

## 1 Introduction

Three species of *Ancylostoma* hookworms, namely *Ancylostoma tubaeforme, Ancylostoma braziliense* and *Ancylostoma ceylanicum*, are common parasites of cats with variable prevalence depending on the geographical region. *Uncinaria stenocephala*, a typical hookworm of canids in temperate regions, has been rarely reported in cats (Bowman et al., 2002). In addition, there are reports on the occurrence in cats of the canine hookworm *A. caninum* (Baker et al., 1989; Coelho et al., 2011). *Ancylostoma ceylanicum*, unlike the other hookworms infecting cats and dogs, may cause patent infection in humans and therefore impose a remarkable zoonotic risk in endemic areas. It is widespread in subtropical and tropical South East Asia and Pacific region and has also been reported from the Arab Peninsula, southern Africa and South America (Bowman et al., 2002; Traub, 2013).

Like other hookworms, *A. ceylanicum* has a direct life cycle, and infection is acquired by oral ingestion or percutaneous penetration of infective third-stage larvae. The parasitic life cycle of *A. ceylanicum* has been studied in detail in puppies (Yoshida et al., 1974): fourth stage larvae were seen within the intestinal wall 2–3 days after oral infection, first immature adults (fifth-stage nematodes) were present in the intestinal lumen as early as 6 days post infection, together with late fourth-stage larvae, and first eggs were passed in the feces 14 days after infection. The development of *A. ceylanicum* in cats has not been studied in detail. However, an early investigation in four kittens until 12 days post infection (Vevers, 1921) and recently conducted work determining a pre-patency period of 14–16 days (Taweethavonsawat et al., 2012, 2013; 2019) indicate that the development of *A. ceylanicum* in cats is the same as that in dogs.

Prevention and timely treatment of infections with *A. ceylanicum* in companion animals is essential to limit the spread in urban environments and to minimize the risk of transmission to humans of this zoonotic parasite.

Broadline® is a broad spectrum parasiticide which comprises the macrocyclic lactone eprinomectin with nematocidal activity combined with fipronil, (*S*)-methoprene and praziquantel. This topical combination product has been confirmed to be efficacious against various gastrointestinal helminths including adult and developing fourth-stage larval (L4) *A. tubaeforme* and adult *A. braziliense* (Prullage et al., 2014). The high level of efficacy against hookworms was confirmed in a field study conducted in Europe (Rehbein et al., 2014).

The study reported here was conducted to evaluate the efficacy of Broadline® in cats against developing L4 and adult *A. ceylanicum* hookworms.

## 2 Material and methods

The study design was based on the International Cooperation on Harmonisation of Technical Requirements for Registration of Veterinary Medicinal Products (VICH) Guidelines 7 and 20 (Vercruysse et al., 2001, 2002), and the “World Association for the Advancement of Veterinary Parasitology (WAAVP) guidelines for evaluating the efficacy of anthelmintics for dogs and cats” (Jacobs et al., 1994).

The study was conducted in accordance with VICH Guideline 9, entitled *Good Clinical Practice*. Personnel involved in the collection of efficacy data were masked as to the assignment of the cats to treatment groups.

### 2.1 Experimental animals

Twenty-four healthy, 22–27 week old purpose-bred European Short Hair cats of equal gender ratio and weighing approximately two to four kg were included in the study. The cats were nematode-naïve and confirmed to be free of patent gastrointestinal nematode infections by standard fecal examination (Wetzel, 1951) prior to inoculation. Cats were housed individually during the study and fed commercial dry food; the environmental conditions were identical for all animals. Study animals were handled with due regard for their welfare and in compliance with the company’s Institutional Animal Care and Use Committee approvals and applicable national regulations.

### 2.2 Experimental design, inoculation, parasite recovery and count

For infection, cats were inoculated orally with approximately 300 third-stage larvae of *A. ceylanicum*. The *A. ceylanicum* isolate was of canine origin from Thailand and was maintained in cats in the laboratory for two years prior to use. No vomiting occurred after inoculation of the cats.

Two days post inoculation [dpi], the cats were ranked by decreasing pre-treatment body weight, formed into eight blocks of three cats each, and were then allocated randomly to serve either as untreated controls (Group 1) or to receive Broadline® when hookworms were in the developing L4 stage of development (5 dpi; Group 2) or were adults (25 dpi; Group 3). Treatments were administered once topically at the minimum recommended dose of 0.12 mL/kg body weight using disposable 1 mL syringes with 1/100 mL graduation, corresponding to 0.48 to 0.49 mg eprinomectin per kg body weight.

Fecal samples were collected 22 dpi and subjected to a standard McMaster technique (Wetzel, 1951) for egg counts. For parasite recovery and count, cats were humanely euthanized 35 dpi, and total gastrointestinal tract contents were washed over a 150 μm mesh-sized sieve and examined for nematodes. Morphology of the hookworms recovered was consistent with the descriptions of *A. ceylanicum* by Biocca (1951).

### 2.3 Data analysis

*Ancylostoma ceylanicum* counts were transformed to the natural logarithm of (count +1) for calculation of group geometric mean counts. Efficacy was determined by calculating the percent efficacy as 100[(C-T)/C], where C was the geometric mean among untreated controls and T was the geometric mean among the treated animals. The log-counts of the treated groups were compared with the log-counts of the control group using a F-test adjusted for the allocation blocks used to randomize the animals to the treatment groups. The mixed procedure in SAS was used for the analysis, with the treatment groups listed as a fixed effect, and the allocation blocks listed as a random effect.

## 3 Results and discussion

Daily observation of the cats throughout the study and hourly observation of the cats for four hours following treatment administration revealed no health problems and no adverse events due to the treatment with Broadline®.

While severe infections of *A. ceylanicum* in dogs have been reported to produce abnormal feces (e. g., loose stool; diarrhea, partly bloody) and anemia (Carroll and Grove, 1984; Tielemans et al., 2017), infections produced from inoculation of approximately 300 *A. ceylanicum* larvae of young, well-nourished cats were, consistent with the observations in the present study, not associated with clinical signs (Taweethavonsawat et al., 2012, 2013; 2019).

Experimental studies indicate that the parasitic life cycle of *A. ceylanicum* is the same in both dogs and cats (Vevers, 1921; Yoshida et al., 1974; Taweethavonsawat et al., 2012, 2013; 2019) and allowed to schedule the treatment against developing L4 nematodes in the cats in this study appropriately. However, comparative analysis of data collected in other studies has shown that male and female *A. ceylanicum* recovered from dogs had a larger size than worms recovered from cats but fecundity and number of eggs produced per female worm per day did not differ significantly between dogs and cats (Rehbein et al., 2016).

Fecal examination at 22 dpi revealed no eggs in the feces of the cats treated with Broadline® when the hookworms were fourth-stage larvae while all cats of the control group and of the group to be treated when the hookworms were adult passed hookworm eggs (range, 100 to 1000 eggs per gram [EPG] and 100–900 EPG, respectively) thus confirming the presence of patent (adult) *A. ceylanicum* infection. The egg counts established were at the lower end of the range of EPG counts reported from other studies where cats were experimentally infected with a similar number of *A. ceylanicum* larvae (Taweethavonsawat et al., 2012, 2013; 2019).

Based on worm counts, the efficacy of a single treatment with Broadline® at the minimum label dose was 100% against both developing L4 and adult *A. ceylanicum* (*p* < 0.001). Thus, treatment with Broadline® of cats when *A. ceylanicum* hookworms were in the developing L4 stage of development or were adult totally eliminated the infectionwhile all cats in the control group were infected (range, 8–35 hookworms; geometric mean count 20.1).

These results are consistent with the level of efficacy of Broadline® treatment found in studies against developing L4 and adult *A. tubaeforme* and adult *A. braziliense* hookworms. The assessment of other products in cats against *A. ceylanicum* was based on fecal egg count reduction only (Taweethavonsawat et al., 2012, 2013; 2019) while the present study tested the efficacy of Broadline® based on worm counts after necropsy and thus provide full compliance with the requirements of VICH GL7 and GL20 for the evaluation of the efficacy of anthelmintics in cats. The results of this study demonstrate that Broadline® delivering eprinomectin at the minimum label dose is highly efficacious not only against adult *A. ceylanicum* hookworms but already during the pre-patent phase of infection while the parasites are developing within the host. This is an important feature because it contributes to a more sustainable prevention of hookworm disease by stopping the infection in the cats before becoming patent and in consequence reducing the environmental contamination with hookworm larvae and the risk of transmission of the infection to other animals and humans.

## 4 Conclusion

In conclusion, results of this study demonstrate that a single treatment with Broadline® at the minimum recommended dose is effective against developing and adult *A. ceylanicum* infections of cats and qualify Broadline® as highly efficacious and easy-to-apply product to mitigate the risk of human hookworm infections posed by cats.

## Disclaimer

This document is provided for scientific purposes only. Any reference to a brand or trademark herein is for informational purposes only and is not intended for commercial purpose or to dilute the rights of the respective owner(s) of the brand(s) or trademark(s).

## Declaration of Competing Interest

The work reported herein was funded by Merial, Inc., GA, USA which is now known as Boehringer Ingelheim Animal Health, GA, USA. All authors were employees or contractors of Merial.

## CRediT authorship contribution statement

**Martin Knaus:** Methodology, Investigation, Writing - original draft. **Piyanan Taweethavonsawat:** Resources, Investigation, Writing - review & editing. **Tara Cheesman:** Writing - review & editing. **Martin Visser:** Investigation. **Steffen Rehbein:** Supervision, Writing - review & editing.
